# Study protocol for a parallel-group randomized controlled multi-center trial evaluating the additional effect of continuous ultrasound bladder monitoring in urotherapy for children with functional daytime urinary incontinence (SENS-U trial)

**DOI:** 10.1186/s13063-022-06600-6

**Published:** 2022-08-13

**Authors:** L. L. de Wall, A. J. Nieuwhof-Leppink, E. H. M. van de Wetering, E. Leijn, M. Trompetter, L. M. O. de Kort, W. F. Feitz, R. Schappin

**Affiliations:** 1 Department of Urology, Division of Pediatric Urology, Radboud University Medical Center, Amalia Children’s Hospital, Geert Grooteplein Zuid 10, Nijmegen, 6500 HB The Netherlands; 2 Department of Medical Psychology and Social Work, Wilhelmina Children’s Hospital Utrecht, Utrecht, The Netherlands; 3 TOP voor Kinderen Practice, Arnhem, The Netherlands; 4 Department of Urology, Isala Clinics, Zwolle, The Netherlands; 5 Department of Urology, University Medical Center Utrecht, Utrecht, The Netherlands; 6 Department of Surgery, University Medical Center Utrecht, Utrecht, The Netherlands; 7 Department of Dermatology, Erasmus MC, Rotterdam, The Netherlands

**Keywords:** Bladder training, Urotherapy, Alarm interventions, Children

## Abstract

**Background:**

Lower urinary tract dysfunction or functional urinary incontinence is a common condition with a prevalence up to 21% between 6 and 8 year-old children. It is associated with an impaired quality of life, lower self-esteem, and social stigmatization. Urotherapy is the first treatment of choice for functional daytime urinary incontinence (DUI) in children. Alarm therapy can be a part of urotherapy as it provides the child adequate feedback on wetting accidents. Current alarm systems notify either at a set interval or give a notification when wetting has already occurred to prompt the child to go to the toilet. These alarms do not teach the child the interpretation of the bladder sensation preceding wetting accidents. A new wearable bladder sensor, the SENS-U, recently became available. This is a relative small, wireless ultrasonic sensor, which continuously monitors bladder filling. The SENS-U is able to provide an alarm at the exact moment voiding is warranted. It facilitates the child to learn the sensation of bladder filling preceding voiding in an easier way, increasing the learning curve throughout treatment. Its additional effect in urotherapy on continence and cost-effectiveness is to be determined.

**Methods/design:**

This is a multi-center clinical superiority parallel-group randomized controlled trial including a total of 480 children. Participants between 6 and 16 years of age with functional DUI in which urotherapy is offered as the next treatment of choice are eligible. Four centers, two academic hospitals, and two general care (peripheral) centers are participating. Participants will be randomized at a 1:1:1 ratio into three groups: urotherapy (care as usual), urotherapy with the SENS-U added for 3 consecutive weeks throughout the training, or urotherapy with a SHAM device for 3 weeks. The primary outcome is number of wetting accidents per week after 3 months of training, compared between the SENS-U and the SHAM device. The magnitude of the placebo effect will be assessed by comparing the results of the SHAM group versus the control (care as usual) group.

**Discussion:**

To our knowledge, this is the first trial studying not only the effect but also the cost-effectiveness of alarm interventions as commonly added in urotherapy.

**Trial registration:**

ISRCTN44345202. Registered on March 2022

**Supplementary Information:**

The online version contains supplementary material available at 10.1186/s13063-022-06600-6.

## Background

Lower urinary tract dysfunction (LUTD; functional incontinence) is a common condition with a prevalence up to 21% in 6–17-year-old children [1, 2]. It is associated with an impaired quality of Life, lower self-esteem, and social stigmatization [3, 4]. Children rate “wetting their pants in class” repeatedly in the top 5 of most stressful life events [5].

According to the International Children’s Continence Society (ICCS), urotherapy is the first treatment of choice for functional urinary incontinence [6]. Urotherapy uses non-pharmacological, non-surgical methods and focuses on behavioral interventions, largely based on cognitive-behavioral psychotherapy. The main aim of urotherapy is to achieve the normalization of the micturition and bowel pattern and to prevent further functional disturbances by repeated training [6]. Studies on the effectiveness of urotherapy report success rates between 40 and 70% [7]. A meta-analytic evaluation of incontinence interventions reported a success rate of urotherapy of approximately 54% within 1 year of treatment (complete dry in 44% and a maximum of one wet episode a week in 10%). The overall reported spontaneous recovery rate in children is 15% per year [7–9].

Additional tools often used in urotherapy are voiding diaries, frequency voiding charts (FVC), uroflowmetry, and alarm interventions [6]. The voiding diary is used as a feedback tool that makes children aware of their voiding behavior. An alarm system gives either feedback when wetting accidents has occurred or notifies the child a set interval to go to the toilet. These systems do not teach the child the interpretation of the bladder sensation that precedes wetting accidents. A new wearable bladder sensor recently became available, the SENS-U. This is a relative small, wireless ultrasonic sensor, which continuously monitors bladder filling and alarms the child when it is time to void. The SENS-U may increase children’s awareness of the sensation of a full bladder. It can be personalized by adjusting the percentage of bladder filling at which it sends an alarm, based on the child’s own bladder capacity and FVC [10–12]. This teaches children which bladder sensation corresponds to a nearly full bladder and when it is time to void.

In current urotherapy, the bladder sensation that corresponds to a full bladder is explained by the urotherapist. Biofeedback on bladder filling with the SENS-U enables children to directly feel what the urotherapist means, thereby inducing less trial-and-error and reducing the number of failing experiences. This could improve urotherapy, as a normally time consuming treatment, with less consultations needed during training and subsequent cost reduction.

Proof-of-concept of the SENS-U and its safety and feasibility have already been established [10–12]. In a pilot study of 15 patients between 6 and 16 years, children responded positively to the notification [12]. Clinical data with small number of patients show promising results with a complete response (100% dry) after only 1 week of training in 33–50% [12]. However, no large clinical randomized trials exist to evaluate its true additional effect in urotherapy.

### Aim and objective

The aim of this study is to investigate whether the SENS-U improves the cost-effectiveness of urotherapy. The objective is to find out the most effective urotherapy modality for children with functional daytime urinary incontinence (DUI). This paper describes the study design and the interventions of a parallel-group randomized trial. We hypothesize a steeper individual learning curve and more cost-effective training period if the SENS-U is added to urotherapy.

## Methods/design

### Study design and setting

This study is multicenter clinical superiority parallel-group randomized controlled trial, designed according to the SPIRIT 2013 checklist (Additional file 1). The final study protocol version 6.0, December 2021, was approved by the Ethics Committee of Arnhem-Nijmegen in the Netherlands (NL 78403.091.21) and registered in ISRCTN44345202 (March 2022).

Participating centers in the Netherlands are the Wilhelmina Children’s Hospital in Utrecht, the Radboudumc Amalia Children’s Hospital in Nijmegen, Isala Clinic in Zwolle, and TOP voor kinderen in Arnhem. The first two are academic referral centers, the latter peripheral centers. The study will be conducted from June 2021 to June 2025. First-, second-, and third-line centers are participating in this study with the intention to select not only therapy-resistant children referred to third-line centers but also children seeking medical attention for functional DUI at the start of their condition.

The study consist of three parallel arms: urotherapy (care as usual), urotherapy with addition of a SENS-U device, and urotherapy with addition of a SHAM device. Outcomes parameters are measured at baseline, after the end of 3-week training with or without SENS-U/SHAM, after 3 months at the end of urotherapy, and 6 months after start of the training. Total study duration is 4 years. Individual study duration for subjects is 6 months.

### Funding

This study is funded by an independent self-governing organization in the Netherlands, ZonMw. The producer of the SENS-U, Novioscan BV, will provide an in kind contribution of all necessary SENS-U and SHAM devices, concomitant adhesives, sonogel, and maintenance for the study period to the participating centers. Novioscan BV has no involvement in the initiation, design, progress, termination of the project, data analyses, or publication.

### Recruitment, inclusion procedure, eligibility criteria, and consent

Children between 6 and 16 years with functional DUI for at least 3 months (including ≥ one episode a month), in which urotherapy is considered a suitable treatment option, are recruited at the outpatient department of the participating centers. Children of all gender, ethnic, and socio-economic backgrounds are eligible. Referring general practitioners, pediatricians, and urotherapists—among other health care providers involved in treatment of LUTD in children—will be informed about the trial in journals, conferences, and using online media including a study web site. Children and/or parents who are not yet under active treatment but still interested in the trial can contact the research team by email. One of the research members will contact parent(s) and invite them to the outpatient department in one of the nearby participating centers. Candidates who fulfil all inclusion criteria will receive oral and written information about the study (Additional file 2). A subsequent appointment within 14 days is planned, to answer any remaining questions. If candidates are willing to participate and after signed informed consent, they are allocated to one of the three study arms and planned for intake and start of their urotherapy.

The exclusion criteria are :

- History of congenital urogenital anomalies except for successfully treated mild infravesical obstruction (meatal stenosis, mild urethral valves) > 3 months before inclusion
- History of neurological underlying disease as the cause of LUTD
- History of Botox treatment for LUTD < 3 months for inclusion
- Recurrent urinary tract infection at the start of the study
- Untreated or treated but persisting functional constipation according to Rome IV criteria at the start of the study < 6 months before inclusion
- Recurrent culture proven UTI (less than 3 months before start of the study or not under control by prophylactic antibiotics)
- Previous urotherapy/bladder training within 6 months of start of the study
- Obesity preventing accurate measurement by the SENS-U as defined as a BMI > 95th percentile according to age/gender
- Skin problems in the suprapubic area that are incompatible with the SENS-U adhesive
- Developmental and intellectual disabilities or severe behavioral and social problems that are incompatible with protocolled urotherapy treatment based on the history and opinion of the clinician/urotherapist

### Urotherapy/bladder training

All subjects will receive urotherapy which starts with explanation and instructions. These instructions are also handed out in paper. At baseline, uroflowmetry and ultrasound to measure post void residual (PVR) is performed at least twice. Several patient-related outcome measurements (PROMS) are collected: a 48-h frequency voiding chart (FVC), questionnaires (Pediatric Incontinence Quality of Life (PINQ), EQ-5D-Y), and Rome IV criteria. Number and severity of wet days per week is assessed as baseline parameter.

After this, urotherapy is given for 12 consecutive weeks according to ICCS standards [6]. During this practice period, counseling is given at frequent follow-up appointments according to the local protocol in each center (at least two contact moments after intake and before follow-up at 3 months). Medication for constipation, prophylactic antibiotics, or medication to suppress bladder overactivity are continued if applicable. After 3 months of training, the urotherapist evaluates the outcome of training and number and severity of urinary incontinence per week is assessed again. Uroflowmetry and ultrasound for PVR and voiding diary/FVC parameters are collected again, and outcomes are discussed with the child and its parents. Questionnaires (PINQ, EQ-5D-Y) are filled in, and Rome IV criteria are determined.

If continence is not achieved (or not sufficiently) after 3 months, alternative treatments are discussed. Another contact moment is planned 6 months after start of the treatment to re-evaluate the status, assess the number and severity of urinary incontinence, and collection of previous mentioned PROMS. This is the end of the study period for participating subjects.

### Interventions for subjects randomized to urotherapy + SENS-U or urotherapy + SHAM

Urotherapy is given as mentioned previously and the same for all participating subjects, including the PROMS. In addition the SENS-U or SHAM is added. A health care provider will administer and install the device and explain parents and child how to apply and use the device. The SENS-U is used to provide biofeedback which teaches the child the feeling that corresponds with the feeling of a full bladder. The alarm can be personalized by adjusting the percentage of bladder filling at which it sends an alarm, based on the child’s own bladder capacity and FVC. The SENS-U is able to monitor the natural bladder filling during regular activity in children, as required for application in urotherapy.

The feeling of a device on the children’s belly could increase awareness of their bladder and might induce a placebo effect. Therefore, we included a placebo group, wearing a SHAM device that alerts independent of bladder filling at a set interval. The SHAM device resembles the traditional timer watch which is currently often used in urotherapy [13]. Therefore, it is not considered an extra burden for children to wear a SHAM device. The SHAM and accompanying instructions for use are identical to the SENS-U. The interval between alarms is randomly chosen between 2 and 3 h to appear realistic. To establish a good learning effect but preventing any dependency of the child on a device, either the SENS-U or SHAM will be worn for a total of 21 consecutive days in the overall 3 months of training. After the period of 21 days, an evaluation is done addressing items like comfort and user friendliness. Before the start of the study, all involved health care professionals are instructed about the use of the SENS-U/ SHAM. The project leader will coordinate instructions and training on the research protocol for the SENS-U study. An overview of the study procedures are shown in Table 1.

**Table 1:**
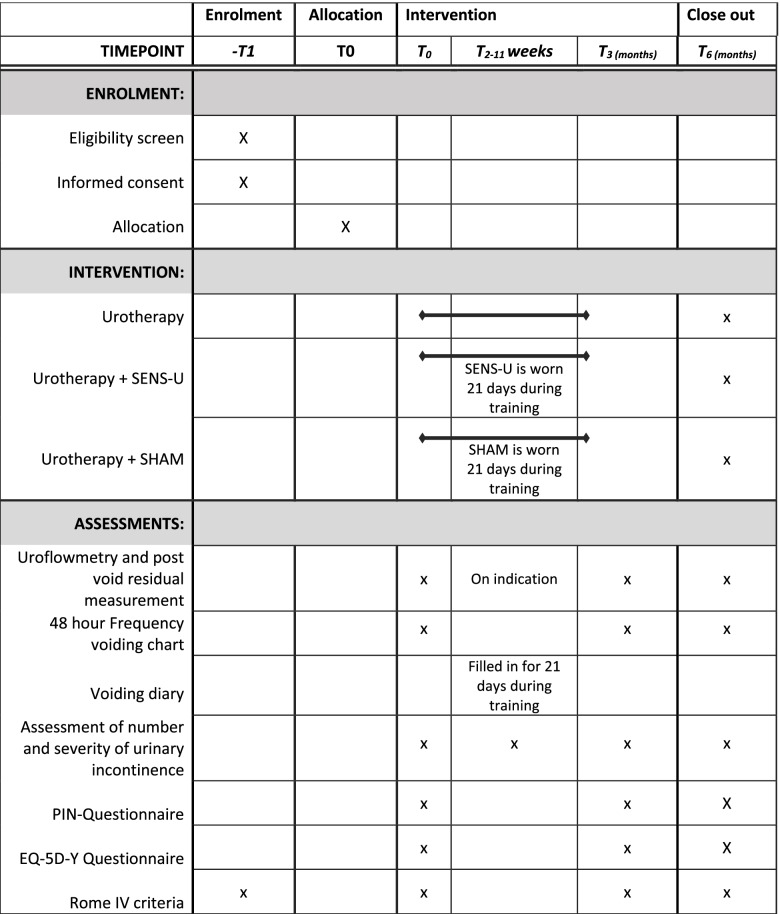
Overview of study procedures

### Randomization

Subjects are randomized to urotherapy for 3 consecutive months with or without 3 weeks use of SENS-U/SHAM device. Permuted block randomization with block size 6 stratified for age groups (6–7 years, 8–10 years, 11–15 years) will be done within each participating center by a computer. The program used is CASTOR EDC. Because some heterogeneity of urotherapy within the participating centers exists (in mode and interval of guidance by the urotherapist), the randomization will be performed within each center to correct for small differences between centers. Subjects are blinded for SENS-U versus SHAM device condition. An independent research member (other than the actual urotherapist treating the subject) is performing the randomization, assigns the device number (either SENS-U/SHAM) to the subject, plans the intake, and administers the device. This is done so outcomes can be attributed to the intervention itself and are only minimally influenced by behavior of the urotherapist who guides the training. The training period is 3 months with use of a device for 3 weeks if randomized to SHAM/ SENS-U during that period. Necessary unblinding during the period of device use (3 weeks) due to urgent medical reasons or unexpected serious adverse advents is very unlikely.

### Primary outcomes and secondary outcomes

The main study outcome is the number of wetting accidents per week after 3 months of urotherapy. Secondary outcomes are number of per-post wetting accidents classified further according to the ICCS standards in complete response (100% reduction of complaints), partial response (50-99% reduction), and no response (less than 50% reduction) [14]. Furthermore, magnitude of the placebo-effect contribution (SHAM versus control) will be assessed next to subjective improvement of symptoms according to child and parents, number of wetting accidents per week during follow-up 6 months after baseline, cost effectiveness, and change in FVC parameters (average, minimum, maximum voided volumes corrected for bladder capacity for age (EBC) between baseline (T0), after 3 months (T3), and after 6 months (T6). Change in disease specific quality of life as measured by the PINQ is also compared between T0, T3, and T6.

Other objectives include adherence to urotherapy and adherence of wearing the SENS-U/SHAM, patient experiences of user friendliness/(dis)comfort of the SENS-U/SHAM device, occurrence of urinary tract infections and constipation, and uroflowmetry curves.

Adherence to urotherapy is defined as number of failed contact moments/number of expected contact moments during the 3 months training. The SHAM and SENS-U measure the number of days actually worn. Adherence of wearing the SENS-U/SHAM is measured as a proportion (number of days not worn/total number of days used in training).

### Sample size

The sample size calculation is based on detecting a difference between the intervention (SENS-U) group and the placebo (SHAM) group at the 3 month endpoint on the primary outcome number of wetting accidents per week. Following the ICCS guidelines, most published studies categorize the number of “wetting accidents per week” in three categories (complete response, partial response, no response) [14]. Information about the initial data used for the categorization (i.e., the number of wetting accidents) is seldom available. Therefore, our sample size calculation is based on a Fisher’s exact test with the known proportions. This will give a slight overestimation of the necessary sample size when analyzing wetting accidents per week, since this latter variable has more variance.

In this calculation, we assume the SHAM condition to be comparable to urotherapy using currently existing wearable alarm treatments (timer watch, pants alarm) [13, 15]. Success rate vary between 53 and 74%, depending on type of referral center [16, 17]. Since this study includes first- to third-line centers, we use an average success rate of 64% to be the estimate success rate of the SHAM condition in our sample size calculation. In addition, we expect at least a 15% additional effect in the intervention (SENS-U) group, based on current clinical experiences with the SENS-U [12].

With a Fisher’s exact test for two independent proportions, one-sided *a* = 0.05, power = 0.80, 64% success in the SHAM group, and 79% success in the SENS-U group, 124 children per group are needed. The one-sided alpha will provide a slight underestimation of the sample size. Given the previous assumption that our sample size calculation result in slight overestimation of the sample size, we presume that the overestimation and underestimation will cancel each other out. With 23% lost to follow-up, we need 160 children per group, 480 children in total.

### Ethical considerations

The study is conducted according to the principles of the Declaration of Helsinki (2013) and in accordance with the Medical Research involving human subjects act (WMO) and other guidelines, regulations, and acts (AGV/WGBO). The study is also conducted according to the codes of conduct for minors (available on the Central Committee on Research Involving Human Subjects (CCMO) site and accepted by the Board of the Dutch Society for Pediatric on May 21, 2001).

There are no known risks or adverse effects to ultrasound imaging, when the intensity is limited to the current Food and Drug Administration (FDA) regulations as is the case in this CE notified device. There are no additional serious risks expected for participants.

Potential burden for the individual subject is mainly time consummation (regular hospital visits/telephone contact with the health care provider) which is not different between the groups and part of standard care in urotherapy. In addition, discomfort or social embarrassment might be experienced by the subjects wearing the SENS-U of SHAM device (despite the fact that only a discrete notification is given). Potential benefit for the individual subject is improvement of their complaints. Whilst it remains unclear if addition of the SENS-U to urotherapy remains (cost)-effective, in the future, other subjects might benefit from the outcomes of this study. Adverse events and serious adverse events will be recorded and managed according to previously mentioned principles, regulations, and guidelines.

Subjects can leave the study at any time for any reason if they wish to do so without any consequences. The investigator can decide to withdraw a subject from the study for urgent medical reasons, like skin problems or severe behavioral/social problems that are incompatible with protocolled urotherapy. Subjects discontinuing the study receive regular follow-up and treatment otherwise for their LUTD.

### Data management

Data from the study participants will be handled confidentially at all times according ICH-GCP regulations. The original signed informed consent forms will be kept in a binder in a locked closet in a locked room of all participating centers. After written consent, per center, each subject will receive a unique identifier, after which members of the research team will extract all necessary clinical parameters from the electronic health records into an electronic case report form (eCRF) of CASTOR EDC. The eCRF contains data items as specified in this research protocol. Access to the eCRF is password protected and specific roles are assigned (e.g., study coordinator, investigator, monitor, etc.). All study protocols, CASTOR generated data, statistical analyses, and reports are stored in a secured Digital Research Environment (AnDREa), and access is password protected and assigned to research members only. At the end of the study, all generated (meta)data will be stored as a proprietary format in a secured data archive called DANS EASY. In order to reproduce the study findings and to help future users to understand and reuse the (meta)data, all changes made to the raw data, including analysis steps, will be documented in an data management analysis plan available on request. Thus, the secure DRE will serve at the end of the study as a data package. More details, including the state of FAIRness, can be found in the data management plan. https://dmponline.dcc.ac.uk/public_plans?page=1&search=additional.

### Data monitoring/auditing

This study is monitored by independent, qualified monitors which are authorized to verify the accuracy and integrity of the data, conduct of the trial, compliance with the protocol, standard operating procedures, good clinical practice, and other regulatory requirements.

They have no competing interests and function independent of the sponsor. According to a predefined monitor plan (available on request) initiation visits, follow-up visits and close out visits are part of the monitoring.

The principal investigators of all participating centers are part of the project management team and meet every 6–8 weeks to discuss the conduct of the trial and are responsible for the correct execution of the study at their site according to the protocol. In addition, the principal investigator of the coordinating center and the project leader have weekly meetings and are responsible for obtaining and maintaining overall authorization of the study and (substantial) amendments to the protocol. The coordinating center accounts for financial affairs and communicates with external parties (Medical Ethical Committee and ZonMw).

This study was funded by an independent self-governing organization (ZonMw) with an independent trial steering committee of 21 members. They were closely involved during the initiation of the project (grant allocation) and will continue to monitor the project till the end. At set intervals, progress reports are required and reviewed. Yearly progress reports are also required and reviewed by the Ethics Committee of Arnhem/Nijmegen.

Day-to-day support is provided by trial offices at each site which help the site specific principal investigator.

### Patient public involvement

A patient advisory board was set up, including parents of each participating center. They were involved from the start. This board gives solicited and unsolicited advice throughout the initiation, design, and progress of the study and implementation of the SENS-U afterwards if the SENS-U turns out to be cost-effective. At least two times a year—and more often if indicated—the board meets with the principal investigator of the coordinating center and the project leader. Reimbursement of costs and deployment are covered.

### Data analysis and publication

Data are analyzed according to an intention to treat analysis, without replacement of individuals who withdraw during the study, using a Poisson or linear mixed model (depending on the distribution of the main outcome) with all available study time-points used. In case of premature withdrawal, last available data will be used. A correction for baseline, center, age, cumulative dose of antimuscarinics (if applicable), and adherence/drop-out will be performed. A random intercept and slope will be estimated if model fit allows for it. Descriptive statistics are used to analyze subjective improvement according to child and parents, adherence to treatment and to the SENS-U/SHAM device, user friendliness and (dis)comfort of the SENS-U/SHAM, and other previously mentioned outcome objectives. The data generated by the validated PIN-Q and EQ-5D-5L instruments are transformed and interpreted according to instructions given for each questionnaire [18–20]. A mixed model analysis is done to detect any difference in outcome between the three groups. Subgroup analysis will be done to study the effect of treatment in different age-groups, center, gender, socio-economic background, and in case of psychiatric co-morbidity. No interim analysis is done as no significant risks or benefits are expected for one of the three different study groups.

The results are shared with relevant fora and data will be presented at international conferences and published in peer reviewed medical journals without restrictions.

### Cost-effectiveness analysis

The economic evaluation is embedded in the design of the clinical study and will be undertaken as a cost-effectiveness analysis with the costs per wetting incident avoided (over a 3 months period) as outcome measure. Based on available evidence it is supposed that the addition of the SENS-U device results in a dominant strategy. If so, no incremental cost-effectiveness ratio can be inferred and cost and effects will be reported separately. Additionally, a cost-utility analysis will be performed with the costs per quality adjusted life-year (QALY) as outcome. Analyses will be performed from a health care perspective (as base-case), and the time horizon is set at 6 months (to make inferences based on sustainability of effect). In case of confounding (baseline differences, etc.), the net monetary benefit approach (NMB) will be applied incorporating the confounders in the regression model with NMB as dependent variable or if dominance occurs the cost and effect outcome. Depending on the efficiency outcome, results will be displayed graphically by means of cost-effectiveness planes and acceptability curves. The Dutch guideline for economic evaluations will be adhered to (ZIN, 2016).

## Discussion

To our knowledge, this is the first trial studying cost-effectiveness of urotherapy and more specifically alarm interventions added. Given the overall prevalence of LUTD, a yearly amount of approximately 8600 children with functional DUI are referred for treatment in the Netherland each year (data retrieved from Statistics Netherlands). Urotherapy as the first treatment of choice is not always sufficient [7]. Additional treatments include medication (mainly off-label or with unwanted side effects), surgery to exclude and treat any type of anatomical anomaly, botulinum toxin, or neuromodulation. Most of these treatment options are either time consuming, expensive, require general anesthesia, or are associated with undesired side effects without the certainty of a successful outcome. As treatment of incontinence is lengthy and therefore difficult to sustain for both children and parents, there is room for improvement of current urotherapy, being the cornerstone of LUTD treatment, because incontinence is a burdensome disease which negatively influences quality of life and self-confidence in children [4]. The SENS-U might help in this process. For professionals, the SENS-U can easily provide data on bladder filling and incontinence during the training and help them to give adequate feedback to the children. For children, the SENS-U can help in the interpretation of the sensation of a full bladder, with subsequent voiding and less urinary incontinence.

If the SENS-U is added to urotherapy, an initial higher complete response of training is expected. An increased number of children is cured in a shorter period of time with less need for other expensive, invasive, and/or time consuming treatments. Furthermore, the possible psychological stress associated with complex medical treatment (like surgery) is prevented.

This study has several strengths. Children from first-, second-, and third-line centers throughout several areas in the Netherlands are included to represent the overall group of children with functional DUI as much as possible. It is one of the few studies that will give a better insight in adherence of alarm systems used, as current literature concerning this element is sparse. As previously mentioned, trials studying not only the effect but also the cost-effectiveness of certain treatment modalities are essential as overall health costs are increasing. The study also has some potential limitations. Complete blinding of subjects is only possible for those allocated to either a SENS-U or SHAM device. Those allocated to urotherapy alone are not blinded (nor is the urotherapist in this case). As cognitive behavioral treatment is an important part of training, motivation in those not randomized to any type of device might be impaired. Potential discomfort or social embarrassment in those allocated to a SENS-U or SHAM might lead to less adherence and subsequent impact on outcome as well.

## Trial status

At the moment, all study centers are finalizing the required procedures to start inclusion. The first patients are included in March 2022. Patient recruitment is planned to be complete in 2024.

## Supplementary Information

**Additional file 1.** SPIRIT 2013 checklist.

**Additional file 2.** Candidates who fulfil all inclusion criteria and receive oral and written information about the study.

## Data Availability

At the end of the study, all generated (meta)data will be stored as a proprietary format in a secured data archive called DANS EASY (https:// easy.dans.knaw.nl). In addition, metadata of published collections can be found through Narcis (https://www.narcis.nl/). Data will be available on request. In line with privacy legislation, it is required that users of these data can be identified (e.g., in case of violation of Data Use Agreement). Therefore, potentially identifiable data are shared under a specific data Use Agreement that requires authentication to download these data sets.

## References

[1] Xing D (2020). Prevalence and risk factors of overactive bladder in Chinese children: a population-based study. Neurourol Urodyn.

[2] Linde JM (2019). Prevalence of urinary incontinence and other lower urinary tract symptoms in children in the Netherlands. J Pediatr Urol.

[3] von Gontard A (2017). Psychological and physical environmental factors in the development of incontinence in adults and children: a comprehensive review. J Wound Ostomy Continence Nurs.

[4] Natale N (2009). Quality of life and self-esteem for children with urinary urge incontinence and voiding postponement. J Urol.

[5] Yamamoto K (1996). Across six nations: stressful events in the lives of children. Child Psychiatry Hum Dev.

[6] Nieuwhof-Leppink AJ (2021). Definitions, indications and practice of urotherapy in children and adolescents: - a standardization document of the International Children’s Continence Society (ICCS). J Pediatr Urol..

[7] Chang SJ (2017). Treatment of daytime urinary incontinence: a standardization document from the International Children's Continence Society. Neurourol Urodyn.

[8] Schafer SK (2018). Standard urotherapy as first-line intervention for daytime incontinence: a meta-analysis. Eur Child Adolesc Psychiatry.

[9] van Gool JD (2014). Multi-center randomized controlled trial of cognitive treatment, placebo, oxybutynin, bladder training, and pelvic floor training in children with functional urinary incontinence. Neurourol Urodyn.

[10] van Leuteren PG (2017). URIKA, continuous ultrasound monitoring for the detection of a full bladder in children with dysfunctional voiding: a feasibility study. Biomed Phys Eng Express.

[11] van Leuteren PG (2018). SENS-U: validation of a wearable ultrasonic bladder monitor in children during urodynamic studies. J Pediatr Urol.

[12] van Leuteren PG, Nieuwhof-Leppink AJ, Dik P (2019). SENS-U: clinical evaluation of a full-bladder notification - a pilot study. J Pediatr Urol.

[13] Hagstroem S (2010). Timer watch assisted urotherapy in children: a randomized controlled trial. J Urol.

[14] Austin PF (2016). The standardization of terminology of lower urinary tract function in children and adolescents: update report from the standardization committee of the International Children’s Continence Society. Neurourol Urodyn.

[15] Halliday S, Meadow SR, Berg I (1987). Successful management of daytime enuresis using alarm procedures: a randomly controlled trial. Arch Dis Child.

[16] de Wall LL, Nieuwhof-Leppink AJ, Schappin R (2021). Alarm-assisted urotherapy for daytime urinary incontinence in children. A meta-analysis.

[17] Meijer EF (2015). Central inhibition of refractory overactive bladder complaints, results of an inpatient training program. J Pediatr Urol.

[18] Versteegh MM (2016). Dutch tariff for the five-level version of EQ-5D. Value Health.

[19] Varni JW, Seid M, Kurtin PS (2001). PedsQL 4.0: reliability and validity of the Pediatric Quality of Life Inventory version 4.0 generic core scales in healthy and patient populations. Med Care.

[20] Bower WF (2006). PinQ: a valid, reliable and reproducible quality-of-life measure in children with bladder dysfunction. J Pediatr Urol.

