# Supplementary material for: Study protocol for a parallel-group randomized controlled multi-center trial evaluating the additional effect of continuous ultrasound bladder monitoring in urotherapy for children with functional daytime urinary incontinence (SENS-U trial)
Source: Trials. 2022 Aug 13;23:648. doi: 10.1186/s13063-022-06600-6 (PMC9375366; doi:10.1186/s13063-022-06600-6)
Supplement: Supplementary file 2 — Additional file 2. Candidates who fulfil all inclusion criteria and receive oral and written information about the study. [file 13063_2022_6600_MOESM2_ESM.pdf]

Logo centrum

Proefpersonen informatie wetenschappelijk onderzoek- ouders

“Studie naar de SENS-U bij blaastraining voor kinderen met urine incontinentie overdag ”

Geachte ouder(s)/voogd(en),
Met deze brief willen wij u informeren over het wetenschappelijk onderzoek “ Studie naar de SENS-U bij blaastraining voor kinderen met urine incontinentie overdag”. In deze brief staat informatie over het wetenschappelijk onderzoek en wat het voor u en uw kind betekent als u mee doet aan deze studie.

Stel uw vragen

U kunt uw beslissing nemen met de informatie die u in deze informatiebrief vindt. Daarnaast raden we u aan om dit te doen:
- Stel vragen aan de onderzoeker of behandelaar die u deze informatie geeft. De gegevens van de onderzoeker in uw centrum vindt u in Bijlage A.
- Praat met uw partner, familie of vrienden over dit onderzoek.
- Stel vragen aan de onafhankelijk deskundige, Dr Lilien, de gegevens staan in Bijlage A
- Lees de informatie op www.rijksoverheid.nl/mensenonderzoek.

Verdere informatie over meedoen aan onderzoek staat in de digitale brochure ‘Medisch-wetenschappelijk onderzoek’.
https://www.rijksoverheid.nl/onderwerpen/medisch-wetenschappelijk-onderzoek/documenten/brochures/2014/09/01/medisch-wetenschappelijk-onderzoek-algemene-informatie-voor-de-proefpersoon

Waarom vragen wij u?

U bent met uw kind op de polikliniek geweest omdat uw kind plas- en/of blaasproblemen heeft. Uw behandelaar heeft besloten dat uw kind blaastraining (urotherapie) gaat krijgen. Als uw kind aan de blaastraining begint is hij/zij tussen de 6 en 16 jaar oud. Wij vragen alle kinderen van deze leeftijd en hun ouder(s)/ voogd(en) om mee te doen aan deze studie. In dit wetenschappelijk onderzoek zijn 4 medische centra betrokken en in het totaal gaan er 480 kinderen meedoen.
Deelnemende centra:

- Radboudumc Amalia Kinderziekenhuis, Nijmegen
- Wilhelmina Kinderziekenhuis, Utrecht
- Isala, Zwolle
- TOP voor kinderen, Arnhem en Zevenaar

Waar gaat het onderzoek over?

Deze studie onderzoekt of de uitkomst van blaastraining beter wordt als de SENS-U wordt gebruikt. Ook wordt gekeken of de kosten van de behandeling goedkoper worden met de SENS-U.
De SENS-U is een klein kastje dat met een pleister op de buik van een kind wordt geplakt. In dit kastje zit een echo die voortdurend meet hoeveel plas er in de blaas van het kind zit. Als de blaas zo vol is dat het kind bijna moet plassen gaat het kastje trillen. Dit kan helpen om kinderen te leren hoe een blaas voelt die zo vol is dat het moet gaan plassen. De SENS-U is anders dan andere apparaten omdat hij al een seintje kan geven vóórdat het kind druppeltjes plas verliest. Wij willen graag onderzoeken of blaastraining met de SENS-U zorgt dat er meer kinderen droog worden. Om dit goed te kunnen onderzoeken worden in de studie drie groepen kinderen vergeleken:

- Kinderen die een blaastraining krijgen.
- Kinderen die een blaastraining met de SENS-U krijgen.
- Kinderen die een blaastraining met een placebo SENS-U krijgen. Placebo betekent nep. Dit is een apparaat dat precies op de SENS-U lijkt en ook een trilsignaal geeft. Anders dan de echte SENS-U trilt de placebo op allerlei momenten en niet precies als de blaas bijna vol is. U weet niet of u de echte SENS-U of de placebo meekrijgt.

Wat betekent meedoen aan deze studie voor u?

U mag zelf kiezen of uw kind mee doet aan het onderzoek. Als u kind meedoet, wordt er geloot in welke groep uw kind komt. U weet dus niet van te voren of uw kind met de SENS-U gaat trainen, met de placebo SENS-U of zonder apparaat. U mag het apparaat lenen van het centrum waar uw kind blaastraining krijgt. De blaastraining zelf is niet anders door deelname aan de studie. Een aantal handelingen zijn wel extra als u meedoet aan de studie.

Hieronder staan de extra handelingen die onderdeel zijn van de studie

- Al u meedoet aan het wetenschappelijk onderzoek wordt er geloot in welke van de 3 groepen uw kind komt.
- Als uw kind geloot heeft voor de SENS-U/ placebo SENS-U krijgt u uitleg en instructies hoe dit te dragen en te gebruiken tijdens de training. De SENS-U/ placebo SENS-U krijgt u 3 weken mee gedurende de training.
- Na de loting en voordat de training begint vragen wij u en uw kind een 3-tal vragenlijsten in te vullen over kwaliteit van leven. Invullen van de vragenlijsten duurt circa 10 minuten.
- 3 maanden na start van de training en 3 maanden na het einde van de training vragen wij u nogmaals de 3-tal vragenlijsten in te vullen over kwaliteit van leven.

3. Welke afspraken maken we met u?

We willen graag dat het onderzoek goed verloopt. Daarom maken we de volgende afspraken met u:

- U komt met uw kind naar iedere afspraak.
- U neemt contact op met de onderzoeker in deze situaties:
  - Uw kind wordt in een ziekenhuis opgenomen of behandeld.
  - Uw kind wilt niet meer meedoen met het onderzoek.
  - Uw telefoonnummer, adres of e-mailadres verandert.

Mogelijke voor-en nadelen en eventueel bijwerkingen / risico's

De SENS-U wordt nog niet veel gebruikt bij blaastraining. We weten dus niet of de SENS-U de uitkomst van blaastraining verbetert. De SENS-U voldoet aan alle veiligheidsvoorschriften en heeft een CE keurmerk. De SENS-U is in het Wilhelmina Kinderziekenhuis onderzocht en is veilig en kan nauwkeurig meten hoe vol de blaas is. Ook meet de SENS-U (of placebo SENS-U) of het wel of niet gedragen wordt.

Mogelijke nadelen, bijwerkingen en risico's kunnen zijn:

- De pleister en gel waarmee de SENS-U op de buik wordt geplakt kan de huid irriteren.
- Kinderen kunnen de SENS-U vervelend vinden zitten.

Gedurende het onderzoek moet u in het totaal op 3 momenten twee vragenlijsten invullen, dat kost maximaal 10 minuten per keer.

De SENS-U of placebo SENS-U kunnen gedragen worden tijdens gymen en of sporten. Mocht uw kind dat niet comfortabel vinden kan het tijdelijk afgedaan worden. De SENS-U of placebo SENS-U is niet waterdicht. Uw kind kan er niet mee zwemmen.

Vrijwillige deelname

Meedoen aan wetenschappelijk onderzoek is altijd helemaal vrijwillig. Het maakt de behandelaar van uw kind niet uit of u wel of niet mee doet, uw kind krijgt altijd goede zorg. Als u na het lezen van deze brief mee wil doen aan het wetenschappelijk onderzoek, kunt u dit laten weten aan de behandelaar van uw kind. Als u niet mee wilt doen hoeft u niets te doen.

Als u niet meer mee wilt doen tijdens de studie

Als uw kind of u **niet** meer aan het wetenschappelijk onderzoek mee willen doen, kunt u dit laten weten aan de behandelaar van uw kind. Uw kind krijgt dan de gebruikelijke behandeling. U hoeft geen reden op te geven waarom u niet meer mee wilt doen.

Wanneer stopt het onderzoek

Het onderzoek duurt 6 maanden. De eerste 3 maanden krijgt uw kind intensieve blaastraining, nadien volgen er nog enkele evaluatie momenten.

Behandeling van persoonlijke gegevens en resultaten

We bewaren deze gegevens:

- de naam van uw kind
- het geslacht van uw kind
- de geboortedatum van uw kind
- (medische) gegevens die we tijdens het onderzoek verzamelen

Waarom verzamelen, gebruiken en bewaren we uw gegevens?

We verzamelen, gebruiken en bewaren uw om de vragen van dit onderzoek te kunnen beantwoorden. En om de resultaten te kunnen publiceren.

De persoonlijke gegevens van uw kind worden pseudoniem voor het wetenschappelijke onderzoek gebruikt. Pseudoniem betekent dat alléén de onderzoekers weten welke gegevens bij uw kind horen. Zij houden dit geheim. Alle andere mensen weten niet welke gegevens er bij uw kind horen Sommige instanties, zoals de inspectie voor de gezondheidszorg en de monitor, mogen volgens de wet ook de gegevens van uw kind zien. Dit is zodat zij kunnen controleren dat het onderzoek goed wordt uitgevoerd. Na de studie kunnen de gepubliceerde resultaten van dit onderzoek gebruikt worden door het bedrijf dat de SENS-U levert. Het bedrijf kan nooit de resultaten herleiden naar uw kind.

Hoelang bewaren we de gegevens van uw kind ?

We bewaren de onderzoeksgegevens van uw kind 15 jaar bij de opdrachtgever. De ruwe data die we daarvoor gebruiken betreft de medische status van uw kind. Deze blijven zoals gebruikelijk levenslang inzichtelijk in het medisch dossier.

Wilt u meer weten over de privacy van uw kind?

- Wilt u meer weten over uw rechten bij de verwerking van persoonsgegevens? Kijk dan op www.autoriteitpersoonsgegevens.nl.
- Heeft u vragen over de rechten van u en uw kind? Of heeft u een klacht over de verwerking van zijn of haar persoonsgegevens? Neem dan contact op met degene die verantwoordelijk is voor de verwerking van uw persoonsgegevens in uw centrum. Zie bijlage A.
- Als u klachten heeft over de verwerking van de persoonsgegevens van uw kind, raden we u aan om deze eerst te bespreken met het onderzoeksteam. U kunt ook naar de Functionaris Gegevensbescherming van uw centrum gaan. Of u dient een klacht in bij de Autoriteit Persoonsgegevens. Zie bijlage A.

Wat gebeurt er met de resultaten van het wetenschappelijk onderzoek?

Als u wilt krijgt u een brief met de groepsresultaten van het wetenschappelijk onderzoek. U geeft op het toestemmingsformulier aan of u dit wil hebben. De resultaten van het onderzoek worden ook in wetenschappelijke tijdschriften en op congressen gepubliceerd. Als uit de studie komt dat de SENS-U de uitkomst van blaastraining verbetert, zal dit in de toekomst mogelijk standaard zorg gaan worden.

Krijgt u een vergoeding als u meedoet aan het onderzoek?

U krijgt geen vergoeding voor deelname aan dit onderzoek

Bent u verzekerd tijdens het onderzoek?

Alle proefpersonen zijn verzekerd, zie Bijlage B.

Hoe geeft u toestemming voor het onderzoek?

U kunt eerst rustig nadenken over dit onderzoek. Daarna vertelt u de onderzoeker of u de informatie begrijpt en of uw kind mee wel of niet meedoet. Wilt uw kind meedoen? Dan vult u het toestemmingsformulier in dat u bij deze informatiebrief vindt. U en de onderzoeker krijgen allebei een getekende versie van deze toestemmingsverklaring.

Vragen

Voor vragen of klachten over het onderzoek heeft neem dan eerst contact op met uw behandelaar of de onderzoeker van uw centrum. Als u vragen heeft over het onderzoek die u wilt bespreken met een arts die niet bij de studie is betrokken (een onafhankelijke arts), dan kunt u contact opnemen met Dr. M Lilien op telefoonnummer: 088 755 4554 of mailadres: nefromaazen@umcutrecht.nl

Met vriendelijke groeten,

Het onderzoeksteam.

Logo centrum

Toestemmingsformulier

Onderzoek:

"Studie naar de SENS-U bij blaastraining voor kinderen met urine incontinentie overdag".

We hebben de informatiebrief over de behandeling en het onderzoek gelezen.

Er is voldoende mogelijkheid geweest om aanvullende vragen te kunnen stellen.

Alle vragen zijn genoeg beantwoord.

Er was voldoende tijd om te beslissen voor deelname aan het onderzoek.

Het is bekend dat meedoen helemaal vrijwillig is.

Het is bekend dat we op ieder moment kunnen beslissen om toch niet mee te doen aan het onderzoek. De (resterende) behandelingen worden dan buiten studie verband, maar zonder eventuele SENS-U of SENS-U placebo voortgezet. We hoeven dan geen reden op te geven.

Wilt u in de tabel hieronder ja of nee aankruisen?

Ik geef toestemming om de gegevens van mijn kind te bewaren om dit te gebruiken voor dit onderzoek, zoals in de informatiebrief staat.	Ja ☐	Nee☐
Ik geef toestemming om mijn kind na dit onderzoek te vragen of hij/zij wil meedoen met een vervolgonderzoek.	Ja ☐	Nee☐
Ik word graag geïnformeerd over de resultaten van dit onderzoek op groepsniveau	Ja ☐	Nee☐

De medische gegeven zullen worden verstuurd naar mijn huisarts. Het is bekend dat deelname aan het onderzoek niet mogelijk is als de huisarts niet op de hoogte mag zijn van de medische gegevens omtrent deze behandeling.

Ben je **jonger dan 12 jaar** en wil je deze behandeling en meedoen met het onderzoek, dan moet een van je ouders bij het gesprek zijn, en beide ouders moeten een handtekening zetten om toestemming te geven:

Logo centrum

Naam ouders (blokletters voorletters en achternaam):

………………………………………………………….			……………….…………………………….

Handtekening ouders:

……………………………………………………….			……………………………………………………

Datum en plaats:

……………………………………………………….			……………………………………………………….

Onderstaand deel is voor de onderzoeker

Ik verklaar hierbij dat ik deze proefpersoon volledig heb geïnformeerd over het genoemde onderzoek.

Als er tijdens het onderzoek informatie bekend wordt die de toestemming van de proefpersoon zou kunnen beïnvloeden, dan breng ik hem/haar daarvan tijdig op de hoogte.

Naam onderzoeker (of diens vertegenwoordiger):

Handtekening:			Datum: __ / __ / __		Plaats:……………………………………………………..

Logo centrum

Bijlage A: contactgegevens [centrum specifiek invullen]

Naam/ email / contact gegevens

Onafhankelijk [arts/deskundige: Dr. Lilien. E-mail adres: nefromaazen@umutrecht.nl.
Telefoon nummer: 088-7554554.

Klachten
Als u klachten heeft kunt u dit melden aan de onderzoeker of aan uw behandelaar. Mocht u ontevreden zijn over de gang van zaken bij het onderzoek en een klacht willen indienen dan kunt u contact opnemen met de klachtenbemiddelaars.

Klachtenbemiddeling van uw centrium: [centrum specifieke gegevens invullen]

Gegevensbescherming
Voor meer informatie over uw rechten kan u terecht bij de functionaris voor de gegevensbescherming van uw instelling.
Functionaris voor de Gegevensbescherming van uw instelling: [centrum specifieke gegevens invullen]

Bijlage B: informatie over de verzekering

Voor iedereen die meedoet aan dit onderzoek, heeft het Radboudumc een verzekering afgesloten. De verzekering dekt schade door deelname aan het onderzoek. Dit geldt voor schade tijdens het onderzoek of binnen vier jaar na het einde ervan. Schade moet u binnen die vier jaar aan de verzekeraar hebben gemeld.
De verzekering dekt niet alle schade. Onderaan deze tekst staat in het kort welke schade niet wordt gedekt.
Deze bepalingen staan in het Besluit verplichte verzekering bij medisch-wetenschappelijk onderzoek met mensen. Dit besluit staat op de website van de Centrale Commissie Mensgebonden Onderzoek, www.ccmo.nl (https://www.ccmo.nl/onderzoekers/wet-en-regelgeving-voor-medisch-wetenschappelijk-onderzoek/besluiten-en-regelingen/besluit-verplichte-verzekering-bij-medisch-wetenschappelijk-onderzoek-met-mensen-2015).
Bij schade kunt u direct contact leggen met de verzekeraar (of schaderegelaar).

De verzekeraar van het onderzoek is:

Naam verzekeraar	:	Centramed B.A.
Adres verzekeraar	:	Postbus 7374, 2701 AJ Zoetermeer
Telefoon	:	070-3017070
E-mail	:	schade@centramed.nl
Polisnummer	:	624.100.021

De verzekering biedt een dekking van *€ 650.000* per proefpersoon met een maximum van *€ 5.000.000* voor het hele onderzoek en *€ 7.500.000* voor schade ten gevolge van medisch-wetenschappelijk onderzoek die per verzekeringsjaar wordt gemeld.

De verzekering dekt de volgende schade **niet:**

- Schade door een risico waarover u in de schriftelijke informatie bent ingelicht. Dit geldt niet als het risico zich ernstiger voordoet dan was voorzien of als het risico heel onwaarschijnlijk was.
- Schade aan uw gezondheid die ook zou zijn ontstaan als u niet aan het onderzoek had meegedaan.
- Schade door het niet (volledig) opvolgen van aanwijzingen of instructies.
- Schade aan uw nakomelingen, als gevolg van een negatief effect van het onderzoek op u of uw nakomelingen (tenzij het onderzoek specifiek betrekking heeft op zwangerschap en geboorte).
- Schade door een bestaande behandelmethode bij onderzoek naar bestaande behandelmethoden.

Voor meer informatie neem contact op met de (hoofd)onderzoeker van uw onderzoeksinstelling (contactgegevens in Bijlage A).
